# Two sisters with cardiac‐urogenital syndrome secondary to pathogenic splicing variant in the *MYRF* gene with unaffected parents: A case of gonadal mosaicism?

**DOI:** 10.1002/mgg3.2139

**Published:** 2023-01-25

**Authors:** Katerina Slaba, Marta Jezova, Petra Pokorna, Hana Palova, Jana Tuckova, Jan Papez, Dagmar Prochazkova, Petr Jabandziev, Ondrej Slaby

**Affiliations:** ^1^ Department of Pediatrics, University Hospital Brno, Faculty of Medicine Masaryk University Brno Czech Republic; ^2^ Department of Pathology, University Hospital Brno, Faculty of Medicine Masaryk University Brno Czech Republic; ^3^ Central European Institute of Technology Masaryk University Brno Czech Republic; ^4^ Institute of Medical Genetics and Genomics, University Hospital Brno, Faculty of Medicine Masaryk University Brno Czech Republic; ^5^ Department of Biology, Faculty of Medicine Masaryk University Brno Czech Republic

**Keywords:** cardiac‐urogenital syndrome, familiar occurrence, *MYRF* gene, Scimitar syndrome, whole‐exome sequencing

## Abstract

**Background:**

Cardiac‐urogenital syndrome [MIM # 618280] is a newly described very rare syndrome associated with pathogenic variants in the myelin regulatory factor (*MYRF*) gene that leads to loss of protein function. MYRF is a transcription factor previously associated only with the control of myelin‐related gene expression. However, it is also highly expressed in other tissues and associated with various organ anomalies. The clinical picture is primarily dominated by complex congenital cardiac developmental defects, pulmonary hypoplasia, congenital diaphragmatic hernia, and urogenital malformations.

**Case Presentation:**

We present case reports of two siblings of unrelated parents in whom whole‐exome sequencing was indicated due to familial occurrence of extensive developmental defects. A new, previously undescribed splicing pathogenic variant c.1388+2T>G in the *MYRF* gene has been identified in both patients. Both parents are unaffected, tested negative, and have another healthy daughter. The identical de novo event in siblings suggests gonadal mosaicism, which can mimic recessive inheritance.

**Conclusions:**

To our knowledge, this is the first published case of familial cardiac‐urogenital syndrome indicating gonadal mosaicism.

## Graphical abstract

We described the first published case of familial cardiac‐urogenital syndrome indicating gonadal mosaicism.

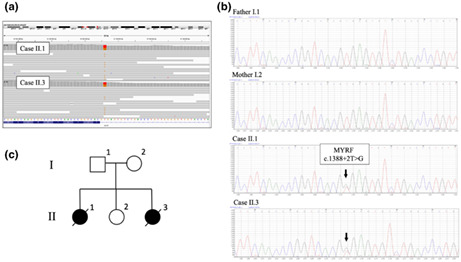

## 1 BACKGROUND

Cardiac‐urogenital syndrome [MIM # 618280] is a rare disease with autosomal dominant inheritance caused by pathogenic variants in the *MYRF* gene that lead to loss of protein function. Together with encephalitis/encephalopathy (MIM # 608329) (Kurahashi et al., 2018), these are the only syndromes in the MIM database associated with MYRF deficiency. It is mainly characterized by complex congenital heart defects, pulmonary hypoplasia, congenital diaphragmatic hernia, and urogenital malformations (Pinz et al., 2018). However, clinical manifestations can be highly variable with varying degrees of severity. To date, more than 30 cases of MYRF deficiency have been described in the literature, but many more patients with similar phenotypic manifestations, including clinically fully expressed cardiac‐urogenital syndrome, have been presented without an established diagnosis by *MYRF* genetic testing (Gupta et al., 2022; Pinz et al., 2018; Qi et al., 2018; Rossetti et al., 2019). So far, no case of familial occurrence associated with gonadal mosaicism has been described.

Haploinsufficiency of the *MYRF* gene is associated with developmental heart defects in up to 94% of patients (Rossetti et al., 2019). The cardiac findings most often included hypoplasia of the left heart (in 44% of patients), infantile Scimitar syndrome with partial or even total anomalous return of the pulmonary veins (31%) (Dupuis et al., 1993; Wang et al., 2018), as well as Fallot's tetralogy, dysplasia or aplasia of the heart valves (aortic, mitral, and tricuspid), and ventricular and atrial septal defect in some patients.

Urogenital anomalies are the second most common structural congenital disability seen in MYRF‐deficient individuals, with approximately 75% affected individuals. Cryptorchidism, micropenis, hypospadias, persistent urachus, and Swyer syndrome have been described in one case (Rossetti et al., 2019). One patient was missing internal genitalia and had a blindly ending vagina (Qi et al., 2018). Approximately two‐thirds of the patients had a diaphragmatic hernia (63%), which led to hepatopulmonary fusion in some patients. Hypoplasia of the right lung has been described in approximately 44% of patients (Rossetti et al., 2019). Other anomalies observed include intestinal malrotation, Meckel's diverticulum, splenic cleft, thymic involution, and thyroid fibrosis (Chitayat et al., 2018; Pinz et al., 2018).

In our report, we present two patients (sisters) who were referred for genetic examination and whole‐exome sequencing (WES) due to the extensive multiple developmental defects of the heart, lungs, and diaphragm; the second patient also had a developmental defect of the urogenital system.

## 2 CASE 1

Family history has been unremarkable so far. The elder of the sisters was born in 2016 to unrelated healthy parents from the first pregnancy, monitored from 27 weeks due to ventricular septal defect, right ventricular hypertrophy, and greater cardiothoracic ratio. Delivery was at 38 weeks gestation after premature amniotic fluid outflow; birth weight was 2700 g (4th percentile) and length was 50 cm (5th percentile). Postpartum adaptation was difficult, developing tachydyspnoea with hyposaturation at around 90%. Slight craniofacial dysmorphism was described—low‐set, backward rotated auricles, markedly wide neck.

The chest X‐ray described a right hemithorax opacification and an undifferentiated right diaphragm. Chest ultrasound revealed relaxation of the right diaphragm with eventration of the liver into the chest, subsequently confirmed by a chest and epigastrium CT scan.

Furthermore, the CT scan described abnormal bronchial branching, hypoplasia of the right lung and partial anomalous pulmonary venous drainage on the right, marked dilatation of the right atrium, and a more spacious right ventricle.

On the day of birth, surgery was performed under general anesthesia—plication of the right and then the left half of the diaphragm. According to the echocardiographic examination, high diaphragm on the right side, right‐sided pulmonary veins converging to the inferior vena cava at the level of the diaphragm, dilatation of the right‐sided compartments, and severe pulmonary hypertension with hypoplasia of the right lung and diaphragmatic bilateral dysfunction/paresis (surgical plication performed) were present, tricuspid regurgitation with a large pressure gradient, perimembranous ventricular septal defect of 7 mm, small foramen ovale, patent ductus arteriosus, and predominant left‐to‐right shunt. The patient was referred to the Department of Cardiosurgery for evaluation of the findings. At 11 weeks of age, catheterization closure, two aortopulmonary collaterals from the truncus coeliacus, and abdominal aorta to the right lung were performed. More extensive cardiac surgery was contraindicated because of marked pulmonary hypertension. She died of heart failure at 12 weeks of age.

**FIGURE 1:**
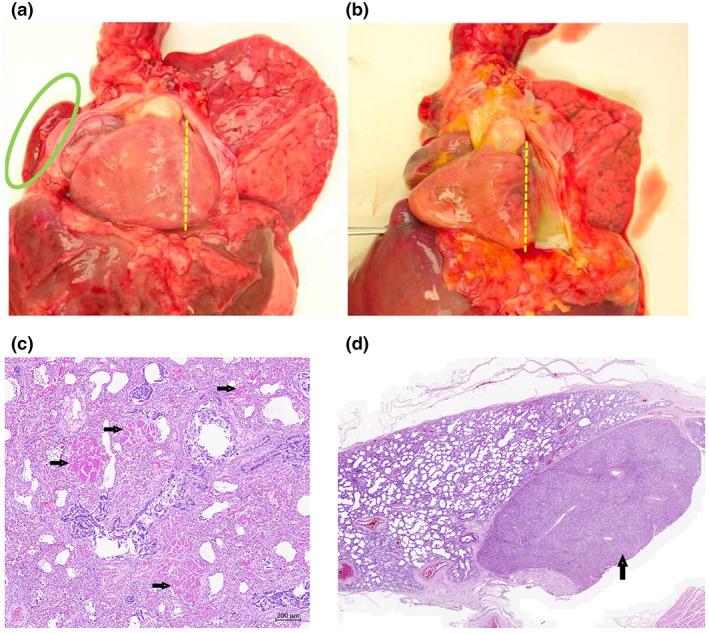
Heart and lung and histological findings. Dextroposition, right lung hypoplasia, and right ventricular hypertrophy were observed in both cases (a) Case 1 and (b) Case 2, indicated by circular and line markers). (c) Rhabdomyomatous dysplasia of the lung observed in Case 1 (indicated by arrows). (d) Hepatopulmonary fusion observed in Case 2 (indicated by arrow).

Autopsy findings (summarized in Table 1) included dextroposition of the heart, partial anomalous return of the right pulmonary veins into the vena cava inferior, perimembranous ventricular septal defect, and a large atrial septal defect as well as, persistent ductus arteriosus, hypoplasia of the right‐sided arteria pulmonalis, and hypoplasia of the right lung (Figure 1a). Hypertrophy of the right heart corresponded to clinically described pulmonary hypertension, the manifestations of which were also detected microscopically (grade 2 according to Heath‐Edwards). The lungs were bilateral unilobar. A rare rhabdomyomatous dysplasia of the right lung was found (Figure 1c). In addition, histologically described traces of dispersed myocardial fibrosis, especially subendocardial, were found. Besides, multiple anomalies of internal organs were found—Meckel's diverticulum of ileum and accessory spleen. The girl did not exhibit malformations of the urogenital system. The diagnosis was concluded as Scimitar syndrome, infantile type.

**TABLE 1:** Summary of autopsy findings

Autopsy findings	Case 1 *2016	Case 2 *2020
	3 months	10 days
Right pulmonary veins	Anomalous drainage to inferior vena cava, above the diaphragm (partial coil embolization performed)	Anomalous drainage—intrahepatic
Right lung	Hypoplasia (21 g)	Extreme hypoplasia (8 g)
	Rhabdomyomatous dysplasia	Hepatopulmonary fusion
Left lung	Unilobar (45 g)	Unilobar (25 g)
Right main pulmonary artery	Hypoplasia	Agenesis
Aortopulmonary collaterals	Branches from thoracic aorta and abdominal aorta/coeliac trunk (coil embolization)	Branches from thoracic aorta
Heart position in the chest	Dextroposition	Dextroposition
Heart defect	PDA	Aortic coarctation
	Large ASD	Hypoplastic aorta
	VSD	Small/stiff left ventricle
		Small ASD
		PDA
Right ventricle	Hypertrophy (pulmonary hypertension grade II Heath‐Edwards)	Hypertrophy
Diaphragm	Bilateral dysfunction/paresis (surgical plication performed)	Right eventration
Urogenital system	—	Double uterus and vagina
		Hypoplastic ovaries
		Splenogonadal fusion (discontinuous type)
Other	Meckel's diverticulum	
	Accessory spleen	

Abbreviations: ASD, atrial septal defect; PDA, patent ductus arteriosus; VSD, ventricular septal defect.

## 3 CASE 2

The younger sister was born 4 years later, in 2020. She was from the third pregnancy that was monitored for aortic coarctation and a smaller left ventricular size. Delivery was at 38 weeks gestation, birth weight of 2960 g (14th percentile), and length of 49 cm (29th percentile). Postpartum adaptation was mildly difficult, with initial oxygen saturation of 70%, spontaneous adjustment after 10 minutes without needing oxygen therapy, and spontaneously ventilated. Prostaglandin E therapy was started for a ductus‐dependent developmental heart defect.

The chest X‐ray again described right‐sided obscuration of the pulmonary wing and undifferentiated contour of the diaphragm, as well as non‐specific spherical obscuration in the central region. The finding was clarified by an angiographic CT scan of the chest, which described an abnormality of the vascular system of the examined area, dominated by a tight coarctation of the aorta, hypoplastic aortic arch, wide‐open ductus arteriosus, aplasia of the right pulmonary artery, hypoplasia of the right lung, anastomoses of the several arterial branches of the truncus coeliacus with the branches of the portal vein, and some branches of the truncus coeliacus pass through the diaphragm into the right lung wing. Additional abdominal sonographic examination showed intrathoracic herniation of the liver and portosystemic shunt. The cardiological examination further revealed a hypoplastic mitral valve and left ventricle. The cardiac defect was assessed as inoperable, and the decision was taken to proceed to palliative care. The patient died of cardiac failure at 10 days of age.

The autopsy findings in the second sister were very similar (see Table 1) and described a complex cardiac defect with dextroposition of the heart and mediastinal shift to the right with extreme right‐sided pulmonary hypoplasia (Figure 1b) with anomalous vascular supply—agenesis of the right branch of the pulmonary artery, aortopulmonary collaterals from the thoracic aorta (and according to angio CT also from the abdominal aorta), and veins with intrahepatic drainage into the intrahepatic part of the vena cava inferior. Furthermore, eventration of the diaphragm and pulmohepatic fusion of the right lung with the liver was described (Figure 1d). Hepatic hemosiderosis was diagnosed. The left lung with anomalous lobation was of adequate size. The cardiac findings showed atrial septal defect, mildly hypoplastic ascending aorta with preductal coarctation, patent ductus arteriosus (post‐prostaglandin E therapy), borderline small left ventricle with hypoplastic papillary muscles, and hypertrophy of the right ventricle, especially its inflow portion. A small pericardial effusion was present. This patient also had a developmental defect in the urogenital system. Specifically, multiple defects of the internal genitalia—hypoplastic ovaries, duplication of the internal genitalia (uterus didelphys and vagina duplex), left‐sided splenogonadal fusion (discontinuous) (Figure S1), and accessory spleen—were also present. These complex autopsy findings led to a suspicion of cardiac‐urogenital syndrome.

## 4 GENETIC TESTING OF BOTH CASES

**FIGURE 2:**
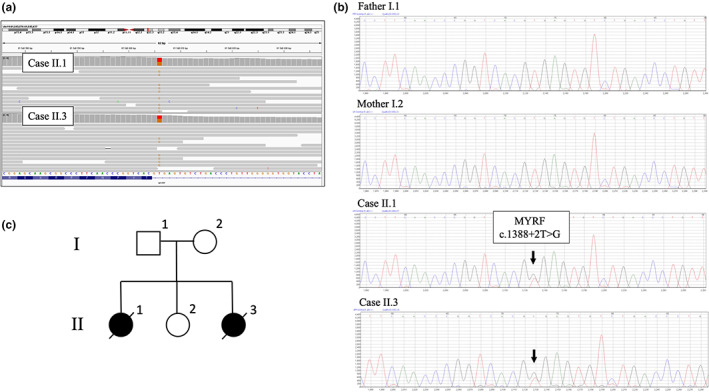
Results of the sequencing analysis of both sisters (case 1 = II.1, case 2 = II.3). (a) Exome sequencing of the patients' DNA presenting pathogenic splicing variant c.1388+2T>G in the MYRF gene was visualized using the Integrative Genomics Viewer software (Broad Institute and the Regents of the University of California, USA). (b) Part of the MYRF gene sequence showing heterozygous pathogenic variant was confirmed to be present in both cases and absent in the parents by Sanger sequencing. (c) The identical de novo events in siblings are consistent with gonadal mosaicism, which can mimic recessive inheritance in the pedigree.

Due to the repeated occurrence of multiple developmental defects in the family, both girls were indicated for genetic examination. Genealogical examination with family history was performed, then normal female karyotypes were confirmed, and DNA microarrays were indicated—all with a negative result. Based on the negative results of the primary genetic examination, both patients were referred to WES after the parents signed the informed consent form. A pathogenic splicing variant c.1388+2T>G was identified in the myelin regulatory factor (*MYRF*) gene (see Figure 2a). The variant is localized to two nucleotides downstream of the boundary between exon 9 and intron 9 and has not previously been described in both clinical or population databases of genetic variants and scientific literature. According to the available splicing prediction algorithms MaxEnt (Yeo & Burge, 2004), NNSPLICE (Reese et al., 1997), and SSF (Shapiro & Senapathy, 1987), there is a 100% probability that the donor splice site is altered, and the abnormally spliced mRNA is produced. The presence of this variant in both patients and its absence in the parents were confirmed by Sanger sequencing (see Figure 2b). The occurrence of identical de novo events in siblings suggests gonadal mosaicism, which can mimic recessive inheritance in the pedigree (see Figure 2c). Non‐paternity was excluded using STR‐based analysis of 17 ENFSI‐recommended STR loci. As the molecular analyses of the affected siblings were carried out postmortem, we were unable to perform RNAseq‐based analysis to assess the variant's effect on splicing due to a lack of suitable biological material.

## 5 DISCUSSION

The *MYRF* gene encodes a transcription factor that autoproteolytically cleaves to directly activate myelin genes (Bujalka et al., 2013; Kim et al., 2021) and as such is essential for myelination of the central nervous system (Emery et al., 2009). Recently, functional mechanisms linking pathogenic mutations in the DNA‐binding domain and intramolecular chaperone auto‐processing (ICA) domain of the *MYRF* gene and birth defects have been described in detail (An et al., 2020; Fan et al., 2021). Variants in the *MYRF* gene have been associated with many diseases as the *MYRF* gene is widely expressed in other tissues such as the stomach, small intestine, and lung (Pinz et al., 2018). Although the function of the MYRF protein outside the nervous system is not well characterized, there is increasing evidence that heterozygous loss‐of‐function variants of *MYRF* can lead to abnormalities in the development of the heart, urogenital tract, diaphragm, and lung (Rossetti et al., 2019). Kurahashi et al. published cases of nine individuals with the same heterozygous variant in the *MYRF* gene who developed encephalopathy and reversible vacuolization of myelin (Kurahashi et al., 2018). In some of these patients with heterozygous loss of function, variants in *MYRF* were also observed as structural abnormalities in various organs, mainly the heart, lung, diaphragm, and genitals (Kurahashi et al., 2018). Since *MYRF* is also expressed in the retinal pigment epithelium, some variants in the *MYRF* gene are associated with ocular disorders such as nanophthalmos (Huang et al., 2021).

The term cardiac‐urogenital syndrome was first used in 2018 in two clinical reports describing three patients with similar phenotypes and *MYRF* gene haploinsufficiency. First, Pinz et al. identified de novo variants in two patients with this disease, and one of these variants (c.2366+1G>A) affected the splice site (Rossetti et al., 2019). Later, Chitayat et al. reported another patient with a heterozygous de novo variant of the *MYRF* gene and a similar phenotype (Chitayat et al., 2018). In the same year, Qi et al. analyzed patients with diaphragmatic herniation and identified a pathogenic variant in the *MYRF* gene in 10 patients with complex cardiopathy and urogenital malformation in a cohort of 362 patients (Qi et al., 2018). In 2020, Alves et al. presented a patient with a confirmed heterozygous pathogenic variant in the *MYRF* gene at 17 years of age with a fully expressed cardiac‐urogenital syndrome. He was further described to have cognitive impairment with an intelligence quotient score of 20. At 4 years of age, brain magnetic resonance imaging showed signs of delayed myelination (Alves & Leão, 2020). The patient's neurological impairment was reported as well by Pinz et al., who described one patient with speech delay who lived until 18 months, and Qi et al., who reported a 2‐year‐old with intellectual disability and motor delay (Pinz et al., 2018; Qi et al., 2018). The prognosis for patients with cardio‐urogenital syndrome is unfavorable, and, in most cases, patients die early in neonatal or early infancy. Three of the reported patients who reached older age (two toddlers and one adolescent) also developed neurological clinical signs with developmental delay.

In the presented cases, there are differences in the autopsy findings of both siblings. In our first patient, among others, a very rare rhabdomyomatous dysplasia of the lung (presence of striated muscle in the lung parenchyma) was described, which is associated with significant malformations involving the heart and lungs. Drut et al. (1988) described the autopsy findings of three neonates with rhabdomyomatous pulmonary dysplasia in great detail (Drut et al., 1988). Other findings described in two of these three patients were phenotypically very similar to the cardiac‐urogenital syndrome. Rhabdomyomatous lung dysplasia is a severe disorder and is probably compatible with long‐term survival only if the histological changes are limited to one lung and if no other life‐limiting malformations are associated (Lienicke et al., 2002).

The occurrence of MYRF‐associated cardiac‐urogenital syndrome has not yet been described in a single family. Based on our findings, it seems that in the familial form of cardiac‐urogenital syndrome, the same pathogenic alteration in the *MYRF* gene can be manifested with variable expressivity. In the first girl, no urogenital anomalies were described in the autopsy findings, and in the second girl, full cardiac‐urogenital syndrome was present. Of the 17 published children with MYRF deficiency, urogenital tract changes were not expressed in a total of 4 children, approximately one‐quarter of the cases (Pinz et al., 2018).

The occurrence of two identical de novo variants observed in our cases is improbable. Both parents are not affected and tested negative. As we excluded non‐paternity, we assume that gonadal mosaicism is involved in this case. Gonadal mosaicism is known to give the impression of autosomal recessive inheritance when recurrence of an autosomal‐dominant condition among offspring of phenotypically normal parents is observed (Figure 2c). Gonadal mosaicism has been extensively documented for several genetic conditions including Duchenne muscular dystrophy, osteogenesis imperfecta, or paternal age‐associated disorders. These reports suggest the need for awareness when discussing the risk of recurrence in clinically and phenotypically normal parents with an affected offspring. The parents had a healthy girl in 2018, cardiological examination showed no pathology, psychomotor development is completely normal, and the girl is doing well. So far, the parents have declined further examination of the daughter, including genetic testing. They have also declined genetic testing of the gametes to confirm the gonadal mosaicism we suspect. The lack of direct confirmation of gonadal mosaicism and lack of WES data from parents, which prevents the possibility of bioinformatic prediction of mosaicism, are the weaknesses of our report.

## 6 CONCLUSIONS

We reported two siblings with postmortem diagnosed cardiac‐urogenital syndrome and elucidated the molecular basis of the disease. To our knowledge, this is the first published case of familial cardiac‐urogenital syndrome with potential gonadal mosaicism. The identical de novo pathogenic event in siblings with unaffected and negatively tested parents suggests gonadal mosaicism mimicking recessive inheritance. The extensive developmental malformations were not compatible with life in both cases. In the case of another planned pregnancy, parents can be offered pre‐implantation genetic testing to increase the probability of having a healthy child.

## AUTHOR CONTRIBUTIONS

KS, MJ, and OS wrote the main manuscript. PP, HP, KS, JP, PJ, MJ, and DP acquired and analyzed the data. PP, HP, KS, JP, MJ, and DP provided interpretation of the data. PJ, DP, JT, and PP commented on the manuscript draft. All authors reviewed the manuscript. All authors read and approved the final manuscript.

## FUNDING INFORMATION

This work was supported by the Ministry of Health, Czech Republic, Conceptual Development of Research Organization (FNBr, 65269705), and the project National Institute for Cancer Research (Programme EXCELES, ID Project No. LX22NPO5102)—Funded by the European Union—Next Generation EU.

## CONFLICT OF INTEREST

The authors declare that they have no competing interests.

## ETHICS APPROVAL AND PATIENT CONSENT

This study was performed according to the Declaration of Helsinki. Written informed consent to participate in the study was obtained from all the participants and/or from parents/legal guardians of the participant. The research was approved by the Medical Ethics Committee of the University Hospital Brno, Brno, Czech Republic.

## Supporting information


Figure S1

## Data Availability

Data are available on request from the authors.
