# Supplementary material for: Two sisters with cardiac‐urogenital syndrome secondary to pathogenic splicing variant in the MYRF gene with unaffected parents: A case of gonadal mosaicism?
Source: Mol Genet Genomic Med. 2023 Jan 25;11(5):e2139. doi: 10.1002/mgg3.2139 (PMC10178803; doi:10.1002/mgg3.2139)
Supplement: Supplementary file 1 — Figure S1 [file MGG3-11-e2139-s001.docx]

**SUPPLEMENTARY DATA**

**Case report**

**The first reported case of familial cardiac-urogenital syndrome associated with the pathogenic splicing variant in the *MYRF* gene**

Katerina Slaba^1^, Marta Jezova^2^, Petra Pokorna^2^, Palova Hana^2^, Jana Tuckova^1^, Jan Papez^1^, Dagmar Prochazkova^1,5^, Petr Jabandziev^1,2^, Ondrej Slaby^3,4*^

*^1^ Department of Pediatrics, University Hospital Brno, Faculty of Medicine, Masaryk University, Brno, Czech Republic;*

*^2^Department of Pathology, University Hospital Brno, Faculty of Medicine, Masaryk University, Brno, Czech Republic;*

*^3^ Central European Institute of Technology, Masaryk University, Brno, Czech Republic;*

*^4^ Department of Biology, Faculty of Medicine, Masaryk University, Brno, Czech Republic;*

*^5^ Institute of Medical Genetics and Genomics, University Hospital Brno, Faculty of Medicine, Masaryk University, Brno, Czech Republic.*

***Corresponding author**

Prof. Ondrej Slaby, PhD

Department of Biology, Faculty of Medicine,

Central European Institute of Technology (CEITEC),

Masaryk University

Kamenice 5

625 00 Brno, Czech Republic

Email: [oslaby@med.muni.cz](mailto:oslaby@med.muni.cz)

**Supplementary data**

**Supplementary Figure 1** Splenogonadal fusion (dissected) observed in Case 2.

**
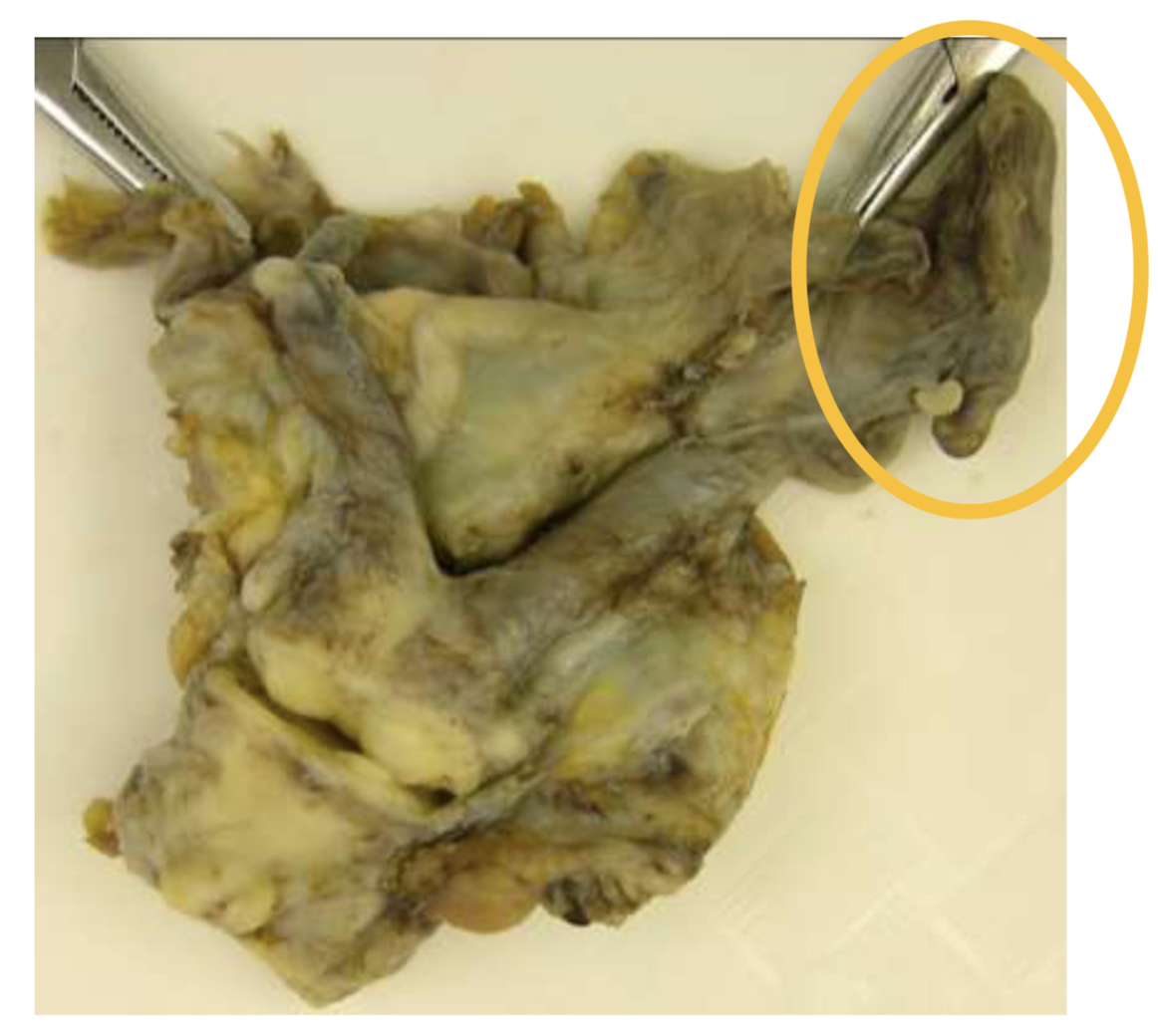
**
